# Stem Cell Therapies for Cerebral Palsy and Autism Spectrum Disorder—A Systematic Review

**DOI:** 10.3390/brainsci11121606

**Published:** 2021-12-03

**Authors:** Justyna Paprocka, Konrad Kaminiów, Sylwia Kozak, Karolina Sztuba, Ewa Emich-Widera

**Affiliations:** 1 Department of Pediatric Neurology, Faculty of Medical Sciences in Katowice, Medical University of Silesia, 40-752 Katowice, Poland; marekwidera@wp.pl; 2 Students’ Scientific Society, Department of Pediatric Neurology, Faculty of Medical Sciences in Katowice, Medical University of Silesia, 40-752 Katowice, Poland; kaminiow.k@gmail.com (K.K.); sylwiakozak@icloud.com (S.K.); k.sztuba96@gmail.com (K.S.)

**Keywords:** stem cell therapy, cerebral palsy, autism spectrum disorder

## Abstract

Autism spectrum disorder (ASD) and cerebral palsy (CP) are some of the most common neurodevelopmental diseases. They have multifactorial origin, which means that each case may manifest differently from the others. In patients with ASD, symptoms associated with deficits in social communication and characteristic, repetitive types of behaviors or interests are predominant, while in patients with CP, motor disability is diagnosed with accompanying cognitive impairment of various degrees. In order to minimize their adverse effects, it is necessary to promptly diagnose and incorporate appropriate management, which can significantly improve patient quality of life. One of the therapeutic possibilities is stem cell therapy, already known from other branches of medicine, with high hopes for safe and effective treatment of these diseases. Undoubtedly, in the future we will have to face the challenges that will arise due to the still existing gaps in knowledge and the heterogeneity of this group of patients. The purpose of this systematic review is to summarize briefly the latest achievements and advances in stem cell therapy for ASD and CP.

## 1. Introduction

With the continuous development of medicine, humankind is gaining new possibilities to treat conditions that until recently could only be treated symptomatically. Autism spectrum disorders and cerebral palsy are among such disorders, which are still a mystery to doctors and scientists. According to ICD-10 (International Classification of Diseases, 10th revision) and DSM-5 (Diagnostic and Statistical Manual of Mental Disorders, 5th edition), autism spectrum disorder is defined as a communication/social interaction disorder with associated repetitive behaviors [1,2]. ASD includes various neurodevelopmental disorders with diverse etiologies such as Autistic Disorder, Pervasive Developmental Disorder not Otherwise Specified (PDD-NOS), and Asperger’s Disorder. The overall estimated prevalence of ASD ranges from 1.5% to 1.8% and an increase has been reported around the world over the past decade [3,4]. Males show a prevalence of 2.8% and females a prevalence of 0.65%, making the male-to-female ratio of 4.3:1 [3,4]. A combination of genetic, environmental and immunological factors underlie ASD [5,6,7,8,9,10,11,12]. It is estimated that up to 1000 potential genes are involved in the genetic determinants of ASD, which are linked by multiple (familial) patterns of inheritance [5,6,7,8,9]. Most of them are responsible for the most essential processes in brain organization and function, such as synaptogenesis, neurotransmitter metabolism, broadly defined neurometabolic, or proper mitochondrial function [13,14,15,16,17,18]. There is also known evidence for alterations in GABAergic circuits in ASD; this evidence comes from postmortem studies showing significantly reduced GAD65/GAD67 levels (two isoforms of glutamic acid decarboxylase which synthesizes the inhibitory neurotransmitter y-aminobutyric acid, GABA) in the parietal cortex and cerebellum and alterations in GABA_A_ and GABA_B_ receptors in postmortem brains of autistic subjects [13,14,15,16,17,18]. These alterations may be the result of widespread changes in GABA innervation and/or release, which may lower the threshold for developing seizures, given the high co-morbidity of ASD and epilepsy [13,14,15,16,17,18]. Moreover, among ASD patients, due to changes in the glutamatergic circuit, there is an increase in excitatory synapse number and spine density. Together, these findings suggest that heterogeneous alterations in glutamatergic and GABAergic systems in the ASD brain may coincide with an overall increased arousal/inhibition ratio, which may manifest as epileptic symptoms, macroscopic brain volume changes, and behavioral changes [13,14,15,16,17,18]. In reference to the close relationship between the immune and nervous systems in the early period of brain development, a hypothesis was developed concerning the origin of disorders in ASD. It assumes a link between neuroinflammation, microglial activation, and/or immune dysregulation in patients with ASD [11,19,20,21,22]. Many risk factors for autism spectrum disorders are known, mainly related to maternal exposure before and during pregnancy. These include exposure to chemicals (e.g., toluene, pesticides), exposure to heavy metals (arsenate, mercury, lead), perinatal trauma, infections during pregnancy, hypoxia, and preterm delivery [10,12,23,24]. In addition to the core symptoms, which restrict range of activities and impairments in social communication, patients with ASD also suffer from concentration disorders, sleep disorders, hyperactivity, motor disorders (e.g., clumsiness or hypotonia), or disorders of normal functioning of the digestive system (chronic constipation and/or diarrhea) [25,26,27]. Current treatments for patients with ASD are limited to psychological interventions, occupational therapy, speech therapy, behavioral therapy, and pharmacotherapy [28,29]. Currently available medications registered for the treatment of patients with ASD target some comorbid conditions but have not been shown to be effective in alleviating or ameliorating core symptoms such as impaired social interaction and communication [22,27,30,31]. In addition, these drugs (e.g., SSRIs, antipsychotics) cause side effects such as extrapyramidal symptoms, sedation, and weight gain [26,32]. All of these significantly reduce the patients’ quality of life. Through functional impairment and the resulting dependence on caregivers and facilities, as well as the lack of therapy aimed at treating the causes of the disorder, the exclusion of ASD patients from society continues to progress.

A very similar situation exists with cerebral palsy. It consists in a group of permanent movement and posture disorders caused by anomalies in the developing brain [33,34,35,36]. These disorders are most often accompanied by sensory, perceptual, cognitive, visual, hearing problems, epilepsy, and musculoskeletal problems [36,37,38,39]. Due to the many problems it generates, cerebral palsy is recognized as one of the most common causes of disability in children [36,40,41]. The overall prevalence of CP is about 2–3 cases per 1000 births, noting that it is significantly higher in children born prematurely (2/1000 births) compared to children born at term (1.1/1000 births) [42,43,44,45,46]. The prevalence rate reaches higher values in developing countries [44,45,47]. The etiology is still unknown, but a complex contribution of genetic, prenatal factors (such as hypoxia, intrauterine growth restriction, or infection) and prematurity is suspected [33,48,49,50]. However, in about 80% of cases, the cause of onset cannot be determined and is considered idiopathic [33,51]. It is suspected that CP patients develop persistent inflammation of the nervous system and subsequent apoptosis, which usually occurs as a result of hypoxia-induced trauma [52]. The current therapeutic management of CP patients requires a multidisciplinary approach that takes into account the medical, social, psychological, and educational needs of the patient [33,36,53]. Treatment includes the use of neurotrophic drugs, physiotherapy, rehabilitation, surgical procedures (such as neurectomy and rhizotomy) and intramuscular injections of botulinum toxin, and other types of symptomatic treatment tailored to the symptoms reported by the patient or their caregivers [33,36,53]. However, despite the many options, the effectiveness of therapies is limited because none of the treatments target brain damage [36,54]. Therefore, new therapeutic options are needed that could repair damaged neural tissues, which would improve patients’ quality of life by improving motor function.

The benefits of therapeutic interventions for individuals with both ASD and CP are limited. In the search for better outcomes in the treatment of these conditions, alternative and complementary therapies are being explored. Due to the involvement of genetic and immunological components in the etiology of these diseases, the need for biological therapy trials targeting the etiology of ASD and CP, especially at the cellular and molecular levels, is indicated. Such opportunities for patients with ASD and CP have been created by the possibility of stem cell therapy. Recent reports suggest that stem cell transplantation results in improvement in several different neurological conditions including stroke, amyotrophic lateral sclerosis, Alzheimer’s disease, spinal cord injury, or Parkinson’s diseases [55,56,57,58,59]. It seems to be a promising option, especially when applied to the brain, a structure generally characterized by slow and limited regeneration. Using stem cells, based on their excellent regenerative abilities, as well as the possibility of differentiation into specific cell types and immunomodulatory effect, we can significantly accelerate the process of repair and remodeling of damaged, immature neurons forming pathological brain structure in ASD and CP patients.

This paper presents a summary of the results of the latest research on stem cell therapies in autism spectrum disorder and cerebral palsy. The collected data were divided into sections, representing types and details of stem cell therapy for each of these disorders.

## 2. Materials and Methods

### 2.1. Search Strategy

A systematic search was conducted in the Pubmed, Medline, and Google Scholar databases to identify the literature related to the stem cell therapies in cerebral palsy and autism spectrum disorder. Three authors independently screened the above-mentioned databases. Each database was searched individually, and search terms were applied line by line and were replicated in every source. The following terms were used in the searching process: “stem cell”, “stem cell therapy” in combination with terms such as “autism”, “autism spectrum disorder”, or “cerebral palsy”. The entire process of searching relevant papers by three reviewers (period for establishing a database of relevant articles) lasted from February 2021 to June 2021, with numerous subsequent updates basedon the latest scientific reports.

### 2.2. Study Selection and Appraisal

Manuscripts were reviewed for titles, abstracts, and entire texts based on the following criteria. The inclusion criteria were as follows: (1) original papers; (2) reviews; (3) stem cell therapy related to autism spectrum disorder or stem cell therapy related to cerebral palsy as a key topic of the paper. The exclusion criteria were as follows: (1) methodological studies, editorials, commentaries, letters, hypotheses; (2) no available abstract; (3) manuscripts in a language other than English. Titles, abstracts, and full-text articles were screened against the inclusion criteria by three reviewers. Next, manual search and reference and citation tracking were undertaken by three reviewers (K.K., S.K., and K.S.) who established the final selection of papers. Any disagreement was resolved by discussion. In the case of no agreement, fourth and fifth independent reviewers made the final decision.

### 2.3. Development of the Review

The analysis was conducted in the following steps. The first step was related to the analysis of selected papers based on titles and abstracts, the second step was connected with the analysis of full-text papers, and the last step included the analysis of the collected data.

## 3. Results

**Figure 1:**
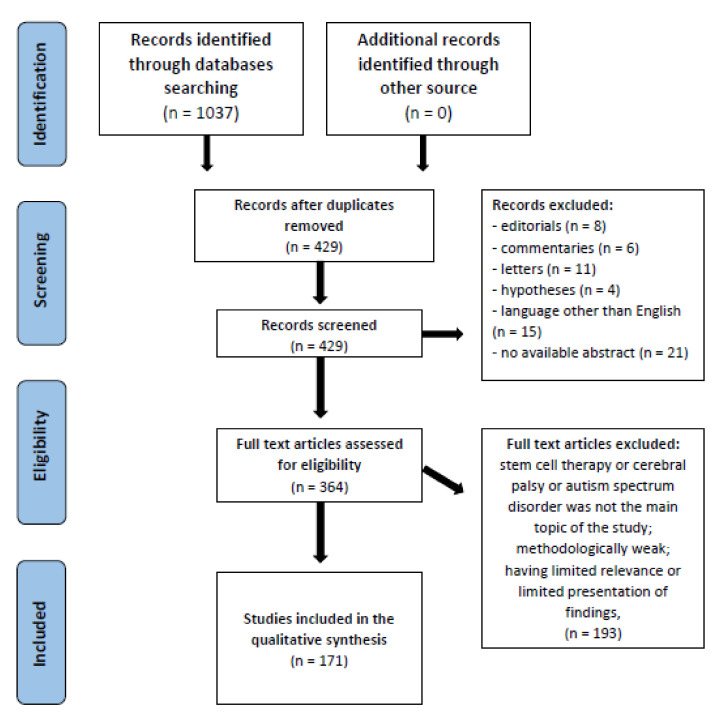
Flow diagram of PRISMA for the strategy research and selection processes for this review.

The preliminary search of the database showed 1037 studies, of which 364 were verified based on the entire manuscript. A total of 171 studies were finally included in the analysis (Figure 1).

### 3.1. Stem Cells Types

Nowadays, many types of stem cells are known, differentiated mainly on the basis of the methods of obtaining them, their origin, and their mechanisms of action. Depending on what we want to achieve, we can use a specific type of stem cells in therapy, corresponding to the profile of action of our goals. In neurological diseases such as ASD or CP, we mainly rely on the use of paracrine effect on host cells, obtained through the influence of substances released from the obtained cells. Thus, host tissue cells are stimulated by cytokines and other mediators for repair processes. Mesenchymal stromal cells and cells from umbilical cord blood are most commonly used due to their good safety profiles, ease of accessibility for their procurement, and high efficacy.

Cord blood cells are among the most easily accessible stem cells. Their collection is a non-invasive method that does not endanger either the mother or her child. While the remaining blood in the umbilical cord is secured, a small amount of blood collected from the mother during delivery is sent for testing to exclude infectious diseases that could infect the recipient during cell transplantation [60]. Umbilical cord blood is an environment rich in diverse stem/progenitor cell populations that may act by different mechanisms that may complement each other, thus improving the effect achieved. In addition to hemopoietic stem and progenitor cells, cord blood also contains many other cells, including mesenchymal stromal cells (MSCs), endothelial progenitor cells, T regulatory cells, and monocyte-derived cells [60,61,62,63]. Cord blood cells exert paracrine effects that promote cell survival, stimulate proliferation and migration of neural stem cells (NSCs), induce regeneration of damaged cells, reduce inflammation, and promote angiogenesis [62,64]. Compared to other types of stem cells, cells from cord blood have many advantages, especially in terms of their safety and efficacy [62,64,65,66], ethical aspects of their procurement [62,67], proliferation without risk of tumorigenesis (carcinogenesis), availability [62,68,69], and regulation of immune response [62,64].

Another rich source of stem cells is bone marrow, but cells harvested from bone marrow have characteristics that distinguish them from those from cord blood. First of all, bone-marrow-derived cells from adults are more immunogenic and are more likely to carry latent viruses that are difficult to detect in assays [62,70]. Moreover, they are characterized by shorter telomeres and lower proliferative potential [62,70]. Bone marrow cells are harvested during a surgical procedure under general anesthesia that takes about an hour. The left or right posterior iliac crest is the most common site of harvest. The iliac crest is preferred for safety reasons because no major blood vessels or organs are located close to this area. A 1–1.5 L mixture of bone marrow and blood is taken from the bone, which regenerates within two weeks.

Mesenchymal stem cells (MSCs) are cells that, according to the International Society of Cellular Therapy (ISCT), are defined by the following minimal set of criteria: grown in adherence to plastic surface of dishes when maintained in standard culture conditions; express cytospecific cell surface markers, that is, CD105, CD90, and CD73, to be negative for other cell surface markers, that is, CD45, CD34, CD14, and CD11b; possess the capacity to differentiate into mesenchymal lineages under appropriate in vitro conditions [71,72]. MSCs show a high expansion potential, genetic stability, stable phenotype, high proliferation rate as adherent cells, and self-renewal capacity and can be easily collected and shipped from the laboratory to the bedside and are compatible with different delivery methods and formulations [72,73]. They can be isolated from many different sources such as bone marrow, cord blood, amniotic fluid, or even adipose tissue [72,74,75]. It is an extremely heterogeneous group of undifferentiated, mulipotent cells that have the ability to differentiate into various cell lineages [71,72,73,74,75]. Although these cells have been shown to mature predominantly into mesodermal tissue cells (such as chondrocytes, osteocytes, or adipocytes), in therapeutic use in CP or ASD, we place more hope on their paracrine abilities, whereby these cells are able to produce factors that activate endogenous restorative mechanisms in damaged tissues, contributing to the restoration of their lost functions [72,76,77,78]. The paracrine action of MSCs is mainly based on their ability to immunomodulate, initiate and promote angiogenesis, support the growth and differentiation of local stem cells, and, importantly, prevent apoptosis and chemoattraction of immune cells [60,72,74,77,79]. MSCs are able to inhibit the release of pro-inflammatory cytokines and block neutrophil recruitment, so, due to their immunosuppressive effects, we can use them for effective autologous treatment as well as for heterologous transplantation, in which they do not require pharmacological immunosuppression [60,72,80]. In addition, they exhibit the ability to modulate humoral immune response as well as cellular by inhibiting the proliferation and maturation of T cells, B cells, NK cells, dendritic cells, and microglia [60]. Due to the low immunogenicity of MSCs, expressed by the low expression of MHC class I molecules and the lack of expression of MHC class II molecules on their surface, these cells can be used completely safely in an allogeneic setting without the need for antigens with matching tissue compatibility between donor and recipient cell leukocytes [60,81]. MSCs differentiate according to signals from surrounding tissues and do not cause uncontrolled growth or tumor formation [60,72,81]. Treatment with MSCs is characterized by high safety as clearly shown in a meta-analysis and systematic review published in 2012, based on 36 studies conducted in 14 countries around the world, involving more than 1000 recipients with various diseases [60,82]. The studies included recipients suffering from cardiovascular, neurological, oncological, or metabolic diseases. Importantly, there were no acute in fusional toxicity organ system complications in response to mesenchymal stromal cell treatment [60,82]. Additionally, there were no treatment-related deaths or malignancies during the 5-year follow-up. The only adverse event was fever, which resolved spontaneously [60,82].

Among many cell types, neural stem cells (NSCs) and neural stem cell-like cells also have their place. These are multipotent stem cells, which have a much more limited ability to differentiate than the previously described cells; that is, they can only differentiate into neurons and glial cells [60,83]. Their actions are responsible, among other things, for the regeneration of nervous tissue by replacing damaged cells, promoting myelination, and secreting neurotrophic substances stimulating neurogenesis [60,81,84]. Their importance in the regeneration of damaged neuronal circuits has also been shown [60,81,84]. These cells are particularly considered as cell therapy material for the treatment of many disorders associated with neuronal or glial cell loss in such clinical entities as stroke, Parkinson’s, Alzheimer’s, and Huntington’s disease; among patients suffering from multiple sclerosis or amyotrophic lateral sclerosis; and among individuals who have suffered spinal cord injuries, among others [60,85,86,87]. However, their extraction from neurogenic areas of the brain is very limited for obvious reasons, so alternative sources are being sought. One such readily available, safe, and ethically uncontroversial source for obtaining cells that can then be differentiated in vitro into neural cells is umbilical cord blood and Wharton’s jelly from the umbilical cord [75,88,89].

Induced pluripotent stem cells (iPSCs) are cells that have undergone in vitro deprogramming, making them capable of differentiating into all cells of the body [81,90]. They can be obtained after applying genetic engineering processes on already differentiated cells. Because of the way they are obtained, iPSCs are not problematic from a practical and ethical point of view; moreover, thanks to the possibility of using allogeneic transplantation, they do not generate anxiety related to its rejection [81,90]. Many ways of obtaining iPSCs are known. They can be divided into two main groups—the first, using viruses as carriers to introduce deprogramming factors (viral-based methods). and the second group, including methods without the use of viruses (non-viral methods) [81,91]. The use of viral vectors (e.g., lentiviruses or retroviruses) allows highly efficient incorporation of the transgene into the host genome and its expression [81,90]. However, this method also carries risks related to the danger of a random site of transgene incorporation and, under unfavorable conditions, the possibility of its reactivation, which increases the possibility of cancer development [81,90]. To minimize these risks, methods unrelated to integration into the host genome can be used, in which adenoviruses, polycistronicepisomal vectors, mRNA, miRNA, or T antigen of SV40 virus and reprogramming proteins are used [81,92,93,94]. Due to the ability of iPSCs to differentiate into neuronal lineage cells, therapy with these stem cells has been shown to be effective in neurological disorders such as Huntington Disease and amyotrophic lateral sclerosis [95,96].

Table 1 provides a brief overview of the stem cell types mentioned above.

### 3.2. Cell Therapies in Autism Spectrum Disorders (ASD)

Stem cell therapy was commonly associated with hematological malignancies; however, in recent decades, it has been also applied to treat other conditions caused by dysfunction of the nervous system such as ASD. This kind of treatment seems to be an attractive therapy: with the enzyme replacement, it prevents the neurological degeneration process as well as extends life in ASD [97,98]. The beneficial effect of stem cell implementation comes from their ability to produce and release the chemokines, cytokines (e.g., anti-inflammatory IL-10 and IL-1Ra), and growth factors (e.g., transforming growth factorβ1, TGF-β1 or granulocyte-macrophage colony-stimulating factor, GM-CSF), which decreases or stops the proinflammation [11,19,20,21,22,72,99].

#### 3.2.1. Stem Cell Mechanism Actions in ASD

The neuropathologies underlying ASD are still not fully known [60]; however, some of them have been already understood (dysfunction of immune system, cerebellum alterations, oxidative stress, hypoperfusion, decreased number of Purkinje cells, defective cortical organization and altered plasticity of dendritic spine morphology, etc.) [100,101]. In Table 2, the stem cell mechanisms of actions in ASD resulting in functional recovery and structural reorganization are provided.

#### 3.2.2. Clinical Studies and the Symptoms Improvement in ASD after Stem Cell Therapy

Currently, there is still a strong need for supporting scientific data in stem cell therapy use in ASD, firstly, to ensure the safety of such treatment and subsequently prove the legitimacy of the use, which will be confirmed by the improvement of the patients’ condition. The necessity for further data collection results from the fact that various stem cell types differ from each other, and this will result in another route of administration (intravenous/intrathecal), dosage, and duration of treatment. Additionally, the time of follow-up needs to be more standardized as only then allows to assess the long-term outcomes and the choice of multiple timely transplantations [81].

A clinical study examining the efficacy of autologous bone marrow mononuclear cell transplantation (BMMNC) in thirty children who fulfilled the autism criteria with Childhood Autism Rating Scale (CARS) scores >37 reported a significant reduction in the severity of ASD with the median CARS score decreasing from 50 (range 40–55.5) to 46.5 (range 33.5–53.5) (*p*  < 0 .05) [108]. Another study assessing the (BMMNC) transplantation infused via the intrathecal route in 32 children with ASD noticed improvements in speech, language patterns, social relationships, and brain metabolism. Out of 32 patients, a total of 29 (91%) patients improved on total Indian Scale for Assessment of Autism (ISAA) scores and 20 patients (62%) showed decreased severity on the Clinical Global Impression Scale (CGI-I scale) and global improvement up to 96% of patients on CGI-II (*p* < 0.001) [109]. According to different research examining the potential in alleviating ASD symptoms by modulating inflammatory processes in the brain using umbilical-cord-blood-derived cell therapy conducted on twenty-five children with a median age of 4.6 years, significant improvements were reported in behavior in the first 6 months post-infusion, which were also sustained at 12 months. It was noted that the improvements were greater in children with higher baseline nonverbal intelligence quotients [110]. The clinical studies mentioned above reported no severe adverse events after cell transplantation and encountered only minor adverse events, such as nausea, vomiting, and pain at the site of injection [108,109,110].

The encouraging results of these clinical trials provide us with future directions for the application of cellular therapy in autism. However, it needs to be emphasized that overall clinical outcome cannot be fully beneficial without the addition of neurorehabilitation such as behavioral and speech therapy, sensory integration, or psychological intervention, etc., which enhances the efficacy of stem cell therapy in ASD.

Table 3 provides a brief overview of clinical trials of stem cell therapies for ASD conducted in recent years.

#### 3.2.3. Limitations

Undoubtedly, stem cell therapy in ASD poses a promising therapeutic option for patients suffering from this condition however, several limitations can be distinguished to establish a final opinion on this treatment. It is worth starting with specifying the conditions for carrying out such therapy, which must be performed according to the clinical guidelines and under laboratory conditions [81]. In addition, there are several other criteria that must be fulfilled that apply especially to the ethical requirements listed in Table 4 [81,113].

Another set of issues concern the insufficient number of studies and subjects included. The samples are not numerous and need to be more standardized, which also makes it difficult to express an unambiguous position. The next debatable point includes reported trials evaluating the efficacy by the use of different scales and scores, which should be preferably standardized as well as internationally validated to ensure the most reliable data. However, dose determination and cell source remain the most challenging. Various studies indicate different effective dosages, which also should be determined with an indication of the cell type therapeutic potential differs according to its source. Considering the limitations mentioned above, large clinical trials are still required to collect more exhaustive data to clearly establish the safety and efficacy of stem cell therapy in ASD.

### 3.3. Stem Cells in Cerebral Palsy Therapy

Cerebral palsy (CP), as mentioned above in the Introduction section, is a group of disorders caused by some insult to the matter of a developing brain. The disorders affect patient’s movement, balance, sensory abilities, and posture. Clinical picture of discussed disease may vary depending on the severity of the damage as well as its location. The vast majority of the cases are connected to perinatal period, whereas only about 8% of cerebral palsy patients have acquired it later on in life [34].

Diagnosis

The diagnosis is made based on five crucial elements:

1. The disease covers a spectrum of symptoms;
2. Though the disorder is permanent, itis not unchangeable;
3. The disorder involves either movement or/and posture problems next to motor function issues;
4. The cause in a non-progressive interference, lesion or abnormality;
5. The cause indicated in point 4 arose in a developing or immature brain. [114]

One should not forget that each CP case is a different individual; therefore, certain inclusion or exclusion criteria may apply.

Gross Motor Classification System

The most commonly used scale in estimating the influence of the disorder on patient’s abilities, and hence their possible improvement after treatment, is Gross Motor Function Classification System, later called GMFCS [36,46,65,115,116,117,118]. It consists of five-level grading system that describes gross motor functions of affected individual [119,120,121]. Levels of Gross Motor Classification System are shown in Table 5.

Gross Motor Function Measure

GMFM- 88 and its shorter version, GMFM-66, are also often come across when delving into the topic of CP. The test checks developmental milestones of a child divided into five categories:

1. Lying and rolling
2. Sitting
3. Crawling and kneeling
4. Standing
5. Walking, running and jumping [122].

#### 3.3.1. Stem Cells

Multidisciplinary care is required to minimize the consequences of cerebral palsy. Aside from long-used therapies, such as intense rehabilitation, spasticity-relieving treatments, sensory and cognitive therapies, and surgical interventions, another innovative approach has been found. Stem cell (SC) therapy has proven promising in various neurological disorders [119]. Surely, scientists’ attention has turned toward CP. Stem cells have been frequently used in several disease in the past years. Taking into consideration that brain damage in CP is non-progressive and usually restricted to a few cell types, one may suspect that stem cells can improve the situation. The fact that another cause of the disease may be demyelination from olygodendrocyte loss suggests this even more, when one remembers that SC have been widely used in the treatment of other diseases based on the same problem [119,123]. The mechanisms in which SCs may be able to help improve the CP patients’ quality of life are due to their regenerative abilities. Once engrafted, the transplanted cells can proliferate. SCs also have anti-inflammatory qualities as they cause a reduction in the number of excitotoxins, cytotoxins, and oxygen free radicals. Their trophic abilities can reestablish balance between neurotrophic factors [65]. There are a lot of stem cell types with promising qualities in the treatment of cerebral palsy [65]. They are enumerated in Table 6.

While a wide range of stem-cell derivation sources are available, the main five sources are predominantly used in attempts to treat patients with CP. These include bone marrow [58,124,125], human umbilical cord blood(hUCB)/umbilical cord (UC) [46,89], fetal brain [117,126], fat, and peripheral blood [52,127]. Stem cells are often derived from an autologous source. However, in children with cerebral palsy, it should be mentioned that autologous bone marrow stem cells are not a good choice as tissue harvesting can cause great physical and psychological trauma to children [56]. Furthermore, in the case of children with cerebral palsy, the fact that all stem cells of allogeneic origin show low immunogenicity, which effectively prevents immune rejection, also gives reasonto abandon the autologous source [67]. It is important to recognize that stem cells from different sources have different efficacy, but interestingly, even stem cells from the same source vary in efficacy during treatment [56]. In 2017, a study was conducted comparing the effectiveness of bone marrow mononuclear stem cells (BMMNCs) and bone marrow MSCs in the treatment of cerebral palsy [42]. This study showed that in children with CP, treatment with bone marrow MSCs was more effective than with BMMNCs [42]. It seems logical that the use of neural stem cells is an ideal option for the treatment of damaged neurons, as well as their extraction from the fetal brain, which is their optimal source, but this solution is still controversial due to ethical issues [56,117,126].

Table 7 provides a brief overview of published clinical trials of stem cell therapies for CP conducted in recent years.

#### 3.3.2. Route of Administration

We distinguish between more and less invasive methods of stem cell administration. The most common are intravenous injection and lumbar puncture [56]. A method using stereotactic brain surgery is also available, but it is less frequently used due to its high invasiveness, by which its side effects are relatively serious, such as damage to blood vessels of the lateral ventricle [128], which can cause brain damage, thus counteracting the therapeutic effects of stem cells [128]. Intravenous administration of stem cells has limited efficacy due to the blood–brain barrier; thus only a small proportion of stem cells can enter the brain parenchyma [56,129]. Thus, regeneration and differentiation of exogenous stem cells in the brain is less efficient [129]. When administered by lumbar puncture, therapeutic agents can reach the brain via the cerebrospinal fluid circulation [130]. Stem cells are also known to be applied via the intranasal route, where the administered cells bypass the blood–brain barrier and enter the brain through the perinuclear space between the somatosensory plate and the olfactory nerve [131,132].

#### 3.3.3. Effectiveness

Research has shown that SC therapy is effective in children with CP, yet it is not spectacular. Children with the first or the second level of GMFCS do not seem to benefit from SC infusion. However, children with severe CP did show an improvement in gross motor skills, according to GMFCS and GMFM-88/66. The dosage of the SC infusion varied from 4 × 10^6^ to 6 × 10^8^. SCs were administered mostly intravenously [36,46,116]. The 2012 research performed an infusion of 8–10 × 10^6^ NPCs in 200 ul normal saline into the lateral ventricle, which caused a major improvement in the first months after receiving treatment. The improvement gradually slowed down; patients did not reach regression. This observation gives an idea of multiple transplants, repeated periodically [117]. Patients aged 3 to 18 years old taking part in a 2018 study received four infusions. According to their GMFM-88 and Comprehensive Functional Assessment, their gross motor and cognitive skills were significantly higher than those in a control group during the while follow-up period, which lasted 24 months, even though both control and researched groups were continually rehabilitated [46]. A study from 2019 suggests that higher dosage correlates with an improved motor outcome [116]. Language improvements have not been observed, but it may be caused by the fact that the treatment has been implemented after the crucial phase in children’s speech development [115]. Language difficulties usually consist of asophia, anarthria, and developmental delays. Children receiving four infusions of hUC-MSCs in a 2020 trial underwent a 12-month-long follow-up and showed a significant improvement in CFA, GMFM, and Activities of Daily Life- ADL. Moreover, their IL-1alpha, IL-6, and TNF- beta were decreased after the transplant, which supports the statement that SCs have anti-inflammatory abilities [36,65]. A peak improvement was noted at six months after the transplant. This study also used an unusual method of monitoring the group’s improvement, as they measured the metabolic activity in the brain. The standard uptake of Fluorine was increased in 3 out of 5 patients. This indicates a recovery in their cerebral metabolic activity based on regional glucose metabolism [36]. According to a case-series of 17 patients, the SC therapy proved effective in 73% of the cases [118].

#### 3.3.4. Safety

The main cause of worries when implementing this treatment is the fear of SCs causing neoplasms in the future. These cells have the ability to induce angiogenesis, which is also a red light when thinking about possible oncogenesis [65,118]. Nevertheless, none of cited papers have mentioned the occurrence of such an adverse event. Reported Severe Adverse Events (SAEs) included infections and seizures coming up equally in both research and control groups. The majority of patients had an uneventful post-injection course or presented mild adverse events such as diarrhea, which proved the therapy’s short-time safety [46,116,118].

#### 3.3.5. Adverse Effects

What scientists always look for, apart from the effectiveness of a given method, are its side effects. In the studies conducted, side effects did occur, but most were mild and transient in nature and were treated symptomatically if necessary [56]. Often, the side effects were related to the way the stem cells were administered, such as pain and redness at the injection site, back pain, and neck stiffness (especially after lumbar puncture) [52,56,58,124,128,133,134,135]. The most common side effects include fever, nausea, vomiting, upper respiratory infections, and diarrhea [52,56,58,124,128,133,134,135]. The most serious side effects were laryngeal stridor and swelling of the tongue [56,134], as well as seizures (however, these patients had already experienced seizures before the stem cell treatment) [124,135]. It is exceptionally interesting that patients who had refractory epilepsy or drug-resistant epilepsy, after being given stem cells as a treatment for cerebral palsy, showed less susceptibility to epileptic seizures than before [136,137]. This interesting lead should prompt researchers and clinicians to further explore the use of stem cell therapy in the treatment of epilepsy.

## 4. Conclusions

It is certain that in the coming years we will witness a gradual expansion of the influence of stem cells in the field of treatment of neurodevelopmental diseases such as cerebral palsy and autism spectrum disorder. It is safe to say that stem cell therapies are a promising novel treatment tool and have potential therapeutic targets based on their excellent regenerative abilities and immunomodulatory effect. Due to the numerous discrepancies in clinical studies concerning the route of administration, cell source, and doses used, it is currently difficult to develop a well-defined consensus of practice in order to maximize therapeutic effect while limiting side effects. Probably one of the most interesting and important aspects is the long-term safety and efficacy of stem cell therapy in ASD and CP. Further studies on the pediatric population are warranted, because still little is known about long-term outcomes and the follow-up period after the current clinical trials is generally short, so the long-term safety and efficacy of stem cell therapy still need to be adequately evaluated.

## Figures and Tables

**Table 1:** Different stem cells, related sources, and their mechanisms of action [60,61,62,63,64,65,66,67,68,69,70,72,73,74,75,76,77,78,81,83,90].

Type of Stem Cells	Source	Mechanism of Action
Fetal stem cells	Fetus, fetal blood, placenta, amniotic membrane, amniotic fluid, umbilical cord	Secretion of neurotrophic factors, immunomodulatory capacities, suppression of proinflammatory processes
Mesenchymal stem cells	Bone marrow, umbilical cord	Paracrine secretion of several anti-inflammatory and survival-promoting molecules (i.e., VEGF, HGF, BDNF, NGF), neuroprotective effects, hypoimmunogenic and immunosuppressive properties
Neural stem cells	Brain (subventricular zone of lateral ventricles and subgranular zone of hippocampus)	Secretion of neurotrophic factors, maintenance of homeostasis, neuroprotective effects, differentiation into neural-type cells
Adipo-derived stem cells	Adipose tissue	Secretion of trophic factors Immunosuppressive and hypoimmunogenic effects
Umbilical cord- and amniotic fluid-derived stem cells	Umbilical cord, placenta, amniotic fluid	In vitro growth capacity, low immunogenicity and immunomodulation properties
Hematopoietic stem cells	Blood, bone marrow, umbilical cord	Paracrine activity
Induced pluripotent stem cells	Any cell type	Differentiation capacity

Abbreviations: VEGF = vascular endothelial growth factor; HGF = hepatocyte growth factor; BDNF = brain-derived neurotrophic factor; NGF = nerve growth factor.

**Table 2:** Stem cell mechanisms of action in ASD [102,103,104,105,106].

Process	Mechanism of Action
Reduction of inflammation	Immune modulation and neuroprotective effects Inhibition of microglial activation and reduction of proinflammatory cytokinesproduction [102,103,106,107]
Restoration of neural connectivity	Modulation of the excitation and inhibition of neurons by controlling the secretion of neurotransmitters [107] Re-establishment of neural connectivity by new synapse formation [10]
Angiogenesis	Reversion hypoxia caused by hypoperfusion in autism Paracrine activity stimulation endogenous cells, promotion of angiogenesis and differentiation of endothelial cells Formation of new blood vessels reverse hypoxia [105]
Antioxidant activity	Reduction of the superoxide production [105]

**Table 3:** Clinical trials of cell therapies in autism spectrum disorder.

Authors	Year	Study Design	Sample	Patient Age (Years)	Cell Source	Route of Administration
Sun et al. [22]	2020	Singlearm,openlabel	12	4–9	AlloUC	i.v.
Dawson et al. [111]	2020	RCT	180	2–7	Autologous or allogeneic CB	i.v.
					Autologous or allogeneic CB	
					Autologous or allogeneic CB	
					Autologous or allogeneic CB	
					Auto or Allo CB	
Riordan et al. [27]	2019	Singlearm,openlabel	20	6–15	Allo UC	i.v.
Chez et al. [112]	2018	RCT	29	2–6	AutoCB	i.v.
Dawson et al. [110]	2017	Singlearm,openlabel	25	2–5	Auto CB	i.v.

Abbreviations: CBMNC = cord blood mononuclear cells; UC-MSC = umbilical cord derived mesenchymal stromal cells; BM-MSC = bone marrow-derived mesenchymal stromal cells; RCT = randomized controlled trial; CB = umbilical cord blood; i.v. = intravenous administration.

**Table 4:** Ethical requirements in ASD stem cell therapy.

Patient-informed consent for treatment
Indications/contraindications for treatment
Documentation of procedure and therapy
Safety and efficacy evaluations
Policy of repeated treatments
Not charging patients for unproven therapies
Basic principles of cell therapy
Policy of repeated treatments
Publishing responsibility

**Table 5:** Gross Motor Classification System.

Level I	The child’s abilities to walk, run, climb the stairs, and jump without any helpthough their balance, coordination, and speed are compromised.
Level II	The child is usually able to walk, sometimes needing some assistance, and needs a railing while climbing the stairs. Difficulties occur while walking a long distance or in challenging surroundings. Gross motor skills are minimal.
Level III	The child can walk with a mobility device. Climbing the stairs requires the use of a railing and some assistance. Forlong distances, a wheelchair is used.
Level IV	The child requires physical assistance and power mobilities. They may be able to walk short distances with appropiate support.
Level V	The child uses a wheelchair in all surroundings. They have problems with positioning and controling movements of their head, trunk, and extremities.

**Table 6:** Stem cell types promising in the treatment of cerebral palsy.

HAECS—amnion epithelial cells
CD34—expressing cells from umbilical cord blood
ES—embryonic stem cells
Fetal stem cells
IPS cells—induced pluripotent stem cells
MSCs—mesenchymal stem cells
MAPCs—multipotent adult progenitor cells
NSCs—neural stem cells
Olfactory ensheathing cells
OPCs—olygodendrocyte progenitor cells
Human UCB—umbilical cord blood

**Table 7:** Clinical trials of cell therapies in CP.

Authors	Year	Study Design	Sample	Patient Age (Years)	Cell Source	Route of Administration
Guetal. [36]	2020	RCT	40	2–12	Allo UC	i.v.
Huang et al. [46]	2018	RCT	56	3–12	Allo CB	i.v.
Liu et al. [42]	2017	RCT	105	6mo–12	Auto BM	Intrathecal
Rah et al. [52]	2017	RCT	57	2–10	Auto PB	i.v.
Sun et al. [41]	2017	RCT	63	1–6	Auto CB	i.v.

Abbreviations: CB-MSC = cord blood-derived mesenchymal stromal cells; BM-MSC = bone marrow-derived mesenchymal stromal cells; BM-MNC = bone marrow mononuclear cells; NPC = neural progenitor cells; CB = umbilical cord blood; PBMNC = peripheral blood mononuclear cells; i.v. = intravenous administration; mo = months.

## Data Availability

Not applicable.
